# Pitfalls in Urinary Diagnosis

**Published:** 1910-02

**Authors:** 


### Pitfalls in Urinary Diagnosis.

This was the subject considered by Dr. Bransford Lewis, of St. Louis, in his annual address read by invitation before the Ohio State Medical Society at Cincinnati, May 6, 1909.

The author first called attention to the enthralling interest of diagnosis as connected with urinary affections and its supreme importance as compared with other features of the study and practice of this work. Shortcomings in diagnosis frequently allow human beings to suffer for years that might, with diagnosis made clear, be healthy and useful citizens all of that time. Cases were cited in illustration. They also illustrated the habit to which some of the profession were addicted of skipping over the real diagnosis altogether and jumping pell-mell into the treatment, so-called, of a case—treatment which consists of giving medicines for the urinary symptoms presenting, which, if effective at all, accomplishes the greatest harm by palliating and masking the evidences being offered by nature as signals of distress that are the most valuable means possible for directing attention to the afflicted organ. The "beautiful specimens" of dilated ureters, sacculated, destroyed kidneys should be looked upon as post-mortem monuments of exalted ignominy. They are usually the result of the withholding of surgery through total disregard of diagnosis.

It was conceded that this was partly due to the relative inaccessibility of the urinary organs as compared with others of the body. But, aside from the fact that obstacles do not justify lax endeavor in medical work, it was pointed out that the means for investigating precisely and comprehensively the whole urinary tract were multifarious, exact and reliable, and the responsibility of having them used was assumed by every physician who accepted a patient for treatment, whether he himself was capable of using them or not.

The relative importance of the several features upon which a diagnosis must rest, the history, symptomatology, semeiology, physical examination, etc., were taken up and compared to the distinct advantage of physical examination for the development of accurate diagnosis.

The pitfalls of diagnosis were considered as they related to the several parts of the urinary tract as follows: A, of the urethra; B, the prostate; C, seminal vesicles; D, bladder; E, the ureters; F, the kidneys.

The individual steps of physical examination as advised by the author were recounted and explained. Among the especial subjects considered were the several features requisite for a diagnosis of urethritis; the sins of omission and commission with respect to urethral stricture, and how to avoid them; the several requisites for diagnosis of prostatic hypertrophy with obstruction, and how to attain them by means of the definite steps advised; those relating to seminal vesiculitis, to bladder stone and to infections of the upper urinary tract. Incidentally the author expressed his entire lack of confidence in staining methods as a mode of differentiating between smegma tubercle and tubercle bacilli, and indicated his idea of the best mode of distinguishing between them—that of excluding smegma bacilli altogether in obtaining the specimen.

Cystoscopy and ureteral catheterization, their successes and their shortcomings, were given due consideration. Direct issue was taken with a number of teachings current on these subjects. The duty of the practitioner in the presence of hæmaturia was considered, the conclusion reached being quite different from that as ordinarily understood. Probably one of the most interesting and practical parts of the address was that relating to renal and ureteral calculi, the X-ray and ureteral catheterization, under which heading the advantages and disadvantages of the several modes of attaining diagnosis were compared and the fallacies of each revealed. None were infallible and none lacking in merit, and all, therefore, were to be employed and given due credit. Cases of mistaken diagnosis, in which the appendix had been removed under the belief that it was the malefactor, when in reality the trouble lay in the right kidney, or ureter, were cited; others in which ureteral calculus was diagnosed, reliance being placed on the findings of the X-ray alone, only to be found wanting at the time of the operation, were mentioned. The fallacies to which catheterization, taken alone and unsupported, might lead were indicated. The proper means of avoiding these several pitfalls were given.

The final conclusion of the author was that if any one lesson were contained in the paper it was the necessity of subjecting to the scrutiny of direct, methodic, scientific investigation rather than futile analysis of symptoms, all obscure or difficult urinary cases, together with the recognition and avoidance of the numerous pitfalls that lay in the path of the unwary in this field of work.—*Exchange.*

